# Impact of inpatient self-efficacy and trust in physicians on inpatient satisfaction with medical services: the mediating role of patient participation in medical decision-making

**DOI:** 10.3389/fpsyg.2024.1364319

**Published:** 2024-08-30

**Authors:** Haixia Wang, Jie Jia, Yafeng Fan, Hanlin Chen, Yi Lou, Xiaohe Wang, Xianhong Huang

**Affiliations:** ^1^Department of Health Policy and Management, School of Public Health, Hangzhou Normal University, Hangzhou, China; ^2^Nursing Laboratory, School of Nursing, Hangzhou Normal University, Hangzhou, China; ^3^Department of Scientific Research, Zhejiang Cancer Hospital, Hangzhou, China

**Keywords:** inpatient satisfaction, trust in physicians, self-efficacy, patient participation, medical decision-making

## Abstract

**Objective:**

Patient satisfaction reflects the social benefits of hospitals and is an important indicator of hospital performance. This study explores the mechanism through which inpatients’ trust in physicians, self-efficacy, and participation in medical decision-making impact their satisfaction with medical services.

**Methods:**

A questionnaire was administered to 814 inpatients in 10 randomly selected tertiary hospitals and 10 randomly selected secondary hospitals in Hangzhou, China. A correlation analysis and hierarchical linear regression were conducted to analyze the factors influencing inpatient satisfaction.

**Results:**

The outcome measures of trust in physicians and participation in medical decision-making behaviors had significant positive effects on inpatient satisfaction.

Trust in physicians was shown to directly influence inpatient satisfaction, while inpatient participation in decision-making partially mediated this relationship. Inpatient participation in medical decision-making fully mediated the relationship between self-efficacy and inpatient satisfaction.

**Conclusion:**

While inpatients were relatively satisfied, there is room for improvement. Healthcare providers should improve patient trust by actively listening to their needs and providing feedback, establishing effective communication mechanisms. Patient self-efficacy can be enhanced through health education, special lectures, and case sharing. Patients should also be encouraged to actively participate in medical decision-making.

**Practical implications:**

Based on inpatient feedback during a preliminary survey, we refined this study’s questionnaire to enhance its feasibility for future research. This article shares key findings for healthcare managers and providers, advising that patient satisfaction can be enhanced through trust, self-efficacy, and participation.

## 1 Introduction

Patient satisfaction is an individual’s assessment of medical services in relation to their expectations, based on their experience when receiving medical care (Qian et al., 2015). According to *The Sixth National Health Services Statistics Survey Report 2018*, the overall satisfaction rate for inpatient services in China increased by 7.8 to 75.0%, compared to 2013 (Centre for Health Statistics and information, 2021; Ling and Qun, 2014). Despite this improvement, there is scope for further progress. Patient satisfaction directly reflects the quality of hospital services. Low satisfaction may lead to patients no longer choosing a certain medical institution or affect the reputation of the medical institution and have other negative impacts, such as patient distrust of the entire medical system. In the context of fostering a harmonious society, patient satisfaction is considered a key indicator to evaluate the government’s oversight of hospitals and the level of social harmony and stability. In the assessment of hospital ratings, patient satisfaction is crucial as part of “social evaluation” (Xia et al., 2015). As China’s medical reform progresses, enhancing patients’ hospitalization experience has become a top priority (Hu et al., 2020). Therefore, identifying the factors that influence patient satisfaction and implementing measures to meet patient expectations are vital.

Patient satisfaction is a multifaceted outcome that is influenced by sociodemographic characteristics, trust in physicians, patient self-efficacy, and participation in decision-making. A study by Yan et al. (2022) found that factors like age, income, and education level were significantly linked to patient satisfaction. Moreover, Xiaohui (2020) found that trust in physicians significantly impacts patient satisfaction. Waters et al. (2016) observed that physician trust was the main driving factor behind patients’ satisfaction with clinical services. Patient self-efficacy is another important factor. Tung and Chang (2009) demonstrated that healthcare professionals providing disease prevention information can improve patient satisfaction. Denny et al. (2017) found that showing patients who suffered strokes educational, stroke-related videos can improve their satisfaction, which was maintained for 30 days after discharge. Furthermore, patient involvement in medical decision-making is also crucial for enhancing satisfaction. Yanhua (2021) showed that the more physicians valued doctor–patient communication and fully considered patient wishes, the higher patient satisfaction became. Suh and Lee (2010) found that patients actively involved in treatment decision-making scenarios and with more opportunities to explain their symptoms tended to have higher satisfaction levels.

The self-determination theory (SDT) is a motivational theory used in medical and health management. It states that individual behavior is driven by three basic psychological needs: autonomy, competence, and relatedness (Hood and Patton, 2022). Meeting these needs results in ideal conditions for personal growth and happiness (Buttitta et al., 2017). In this study, we utilized SDT to investigate how trust in physicians and self-efficacy impact inpatient satisfaction by involving patient participation in medical decision-making. We focused on the importance of patients’ trust in physicians for fulfilling their relational needs, as it promotes a supportive healthcare environment. Patient self-efficacy plays a critical role in meeting their competency needs, as higher self-efficacy helps patients feel more capable and confident in managing their healthcare. Both trust in physicians and self-efficacy are expected to encourage patients’ active involvement in medical decision-making, leading to increased satisfaction with medical services.

Upon reviewing the current literature on patient satisfaction, it is clear that a substantial body of research findings has been accumulated in this area. However, current research has some limitations. Firstly, there is a lack of studies that combine inpatient trust in physicians with patient self-efficacy to explore the underlying factors influencing inpatient satisfaction. Secondly, insufficient focus has been placed on the mediating role of inpatient involvement in medical decision-making. Moreover, the use of SDT in the context of patient satisfaction is limited. This study aims to examine and verify the direct and indirect pathways linking inpatient trust in doctors, self-efficacy, participation in medical decision-making, and satisfaction through empirical research, along with SDT. The theoretical contribution of this study is to enhance the understanding of patient satisfaction and improve the theoretical framework by integrating SDT. This not only offers new empirical evidence for the application of SDT in the medical field but also establishes a comprehensive theoretical framework for future research, leading to a more thorough understanding of how patient satisfaction is formed. From a practical standpoint, given the current issues in medical services, this study suggests specific initiatives to enhance the patient medical experience, potentially mitigating tensions between medical staff and patients, and laying the groundwork for achieving high-quality sustainable development in hospitals.

## 2 Theory and hypotheses

### 2.1 Inpatient trust in physicians and satisfaction with medical services

Trust in physicians refers to the confidence that patients have in the abilities and motivations of physicians during doctor–patient interactions (Shangxin, 2022). This includes patients’ confidence in physicians’ professional skills and the belief that physicians have a comprehensive understanding of patients’ conditions. Studies have confirmed that physician trust is a prerequisite for improving patient satisfaction (Audrain-Pontevia and Menvielle, 2018) and that patients’ trust in healthcare providers positively affects their satisfaction with medical services (Molina et al., 2015). Birkhäuer et al. (2017) found that when patients trusted healthcare professionals, they were more willing to cooperate with treatment, making it easier to control their symptoms and achieve better treatment outcomes, thereby increasing their satisfaction with medical services.

According to Tan et al. (2015), high levels of satisfaction are positively associated with patient trust. Therefore, increasing patient trust helps establish harmonious doctor–patient relationships, which improves patient satisfaction. Shan et al. (2016) found that trust is the most important factor in enhancing patient satisfaction. Patients who did not trust healthcare professionals were more likely to complain about things such as expenses, equipment, procedures, and poor attitudes among healthcare staff (Min and Yao-guang, 2010), which resulted in lower levels of satisfaction with medical services.

Based on the above, we propose the following hypothesis:

*H1*: Inpatients’ trust in physicians positively affects their satisfaction with medical services.

### 2.2 Inpatient self-efficacy and satisfaction with medical services

Patient self-efficacy comprises patients’ confidence in their ability to achieve expected goals during the healthcare process (Jiahui et al., 2019; Zhenhua et al., 2023). This includes their health literacy, perception of disease severity, and information acquisition abilities. Self-efficacy has long been considered a personal resource that protects patients from the direct harmful effects of disease (Steca et al., 2013). Xian et al. (2023) found that patient satisfaction was higher in an observed group that received a self-efficacy intervention (e.g., health education) than in the control group. Providing patients with disease-related information helped improve their understanding of the disease, alleviate their negative emotions, and enhance their problem-solving abilities and confidence, thereby increasing their satisfaction with medical services. Baiardini et al. (2019) emphasized the importance of patients’ perceptions of disease: the more patients know about their disease, the more likely they are to cooperate actively with healthcare professionals and, thus, obtain better care and feel more satisfied. Haijun (2012) showed that the more information inpatients acquired, the better they could cope with the consequences of the disease, which promoted their medical satisfaction. With this in mind, we propose the following hypothesis:

*H2*: Inpatient self-efficacy positively affects patient satisfaction with medical services.

### 2.3 Patient participation in medical decision-making and satisfaction with medical services

Patient participation in medical decision-making happens when physicians discuss various possibilities, such as treatment options, benefits, and side effects, and jointly make the most suitable health decisions for an individual patient (Yingru, 2021). Patients’ participation in medical decision-making improves their satisfaction. Arnetz et al. (2004) found that jointly formulating realistic treatment goals helped patients achieve a sense of goal attainment, increasing their satisfaction with treatment. Jiangyan et al. (2023) showed that patient participation improved patient satisfaction; patients felt respected and acknowledged during the decision-making process, which alleviated negative emotions and promoted satisfaction. According to Alsarhan et al. (2021), involving patients in treatment planning during the lengthy process of dental implantation improved compliance and enhanced patient satisfaction. The above studies indicate that the more actively patients participate in medical decision-making, the higher their satisfaction:

*H3*: Patient participation in medical decision-making has a positive effect on satisfaction with medical services.

### 2.4 Mediating role of inpatient participation in medical decision-making

Patient participation in medical decision-making plays a mediating role in the relationship between their trust in physicians and their satisfaction with medical services. Relatedly, Chughtai and Buckley (2011) found that trust in supervisors prompted employee engagement, which improved employee performance and innovative work. Moreover, trust can facilitate communication and information exchange, promoting shared decision-making between physicians and patients (Obeidat and Lally, 2018) and resulting in greater patient compliance and satisfaction (Nakayama et al., 2020).

Furthermore, patient participation in medical decision-making can mediate their self-efficacy and satisfaction. In the education field, one study showed that student engagement in learning partially mediated the relationship between self-efficacy and learning satisfaction (Xin and Cailan, 2021). Furthermore, hospitalized patients with chronic diseases who were well-informed about their condition demonstrated better skills in managing their health, felt more empowered, and were able to participate in healthcare decision-making, ultimately experiencing improvements in their health and satisfaction (Xue, 2017). This evidence leads us to the following hypotheses:

*H4a*: Inpatient participation in medical decision-making has a positive mediating effect on the relationship between patient trust in physicians and satisfaction with medical services.

*H4b*: Inpatient participation in medical decision-making has a positive mediating effect on the relationship between self-efficacy and satisfaction with medical services.

**Figure 1:**
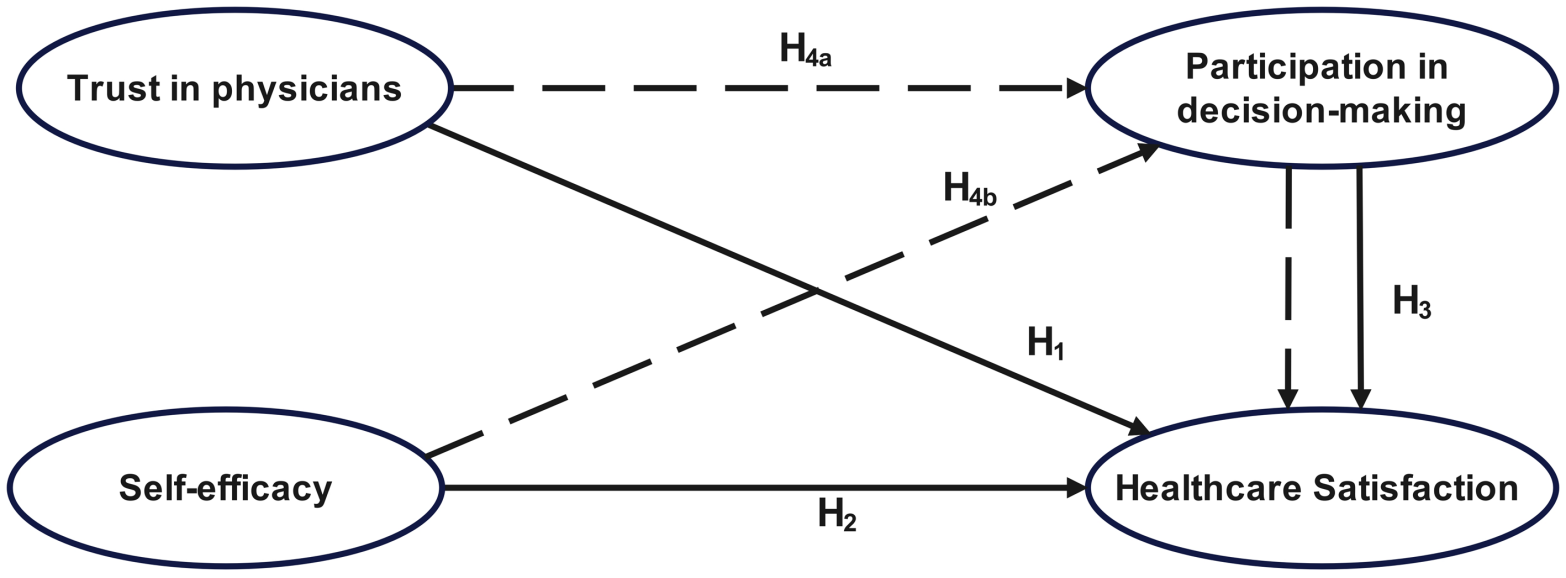
Diagram of theoretical model of inpatient satisfaction with medical services.

Based on the self-determination and self-efficacy theories, this study establishes a theoretical model (Figure 1), with patient trust in physicians and self-efficacy as independent variables, satisfaction with medical services as the dependent variable, and patient participation in medical decision-making as the mediating variable.

## 3 Materials and methods

### 3.1 Participants and procedures

Hangzhou City in eastern China boasts ample healthcare resources, with 138 community health centers, 30 secondary public hospitals, and 22 tertiary public hospitals (Hangzhou Municipal Bureau of Statistics, Hangzhou Socio-economic Survey Team, 2022).

According to Kendell’s sample estimation method (Ming et al., 2024), the sample size should be 10–20 times the number of items in the questionnaire. The questionnaire in this study has 60 items, so the required sample size is between 600 to 1,200 participants. Accounting for an expected non-response rate of around 20%, the adjusted required sample size is between 720 to 1,440 participants. Based on these factors, we initially established the sample size at 900 to guarantee sufficient power and reliability of the findings. A stratified random sampling method was used based on two hospital grades. We selected 10 tertiary and 10 secondary hospitals in Hangzhou. Convenience sampling was then conducted in each hospital, with approximately 30 and 60 people from each secondary and tertiary hospital, respectively, being sampled. The inclusion criteria were: (1) provide informed consent to participate; (2) be 18 years old or older; (3) have a stable medical condition and good mental state; and (4) have the capacity and willingness to participate in the study. The exclusion criteria were: (1) lack of capacity and willingness to participate; (2) presence of mental illness, communication difficulties, or deaf-mutism; and (3) patients in critical care wards. This study was approved by the scientific research ethics committee of Hangzhou Normal University (approval number: 2021–1,146). All procedures were conducted in accordance with the ethical standards of the responsible committee on human experimentation and with the Declaration of Helsinki.

In order to guarantee the effectiveness and feasibility of our study design and the comprehensiveness of the finalized questionnaire, we conducted a preliminary investigation. This phase aimed to assess the clarity and relevance of the survey questions, identify potential challenges that could arise during the actual data collection, and gather experience in the implementation of field survey operations. Five graduate students with relevant fieldwork experience and two researchers performed the final survey from February 1 to April 30, 2021. The investigators received standardized training. Participants were strictly screened according to the inclusion criteria, and one-on-one surveys were conducted. After completion, the questionnaires were organized and coded. Two researchers inputted the data into statistical software to verify its accuracy. A total of 900 questionnaires were distributed to individuals approached for the study. Out of these, 850 completed questionnaires were collected, resulting in a response rate of 94.4%. In total, 50 questionnaires were invalidated because some of the questions were left unanswered. The analysis included 814 eligible questionnaires, for an effective response rate of 95.8%. Supplementary Table S1 lists the characteristics of the 814 participants.

### 3.2 Measures

The questionnaire comprised five parts: demographic characteristics, self-efficacy, trust in physicians, participation in medical decision-making, and satisfaction with medical services. The reliability of the scale was assessed using Cronbach’s α and composite reliability (CR). When both coefficients were above 0.7, the scale demonstrated good reliability. The structural validity was determined by factor load, and convergence validity was measured by calculating average variance extracted (AVE). AVE represents the variance captured by a construct in relation to random measurement error. For a scale to be considered valid, both factor load and AVE values should exceed 0.5. However, AVE values between 0.36 and 0.50 still indicate acceptable convergence validity. The detailed results are provided in Supplementary Table S2.

#### 3.2.1 General information questionnaire

This questionnaire comprised 12 items, including demographic characteristics and medical treatment-related items.

#### 3.2.2 Inpatient self-efficacy scale

This scale is based on Giesler and Weis (2008) scale of patient participation ability. Selected items are categorized into two aspects: participation consciousness and self-evaluation, comprising a total of 8 items. Participation consciousness pertains to patients’ willingness to engage in medical decision-making. It is crucial for patients to be informed about their condition, treatment, and recovery prior to seeking medical assistance, and to actively communicate and collaborate with doctors during the decision-making process. Items like “I can gather information about diseases and treatments from books and the Internet” assess this aspect. Self-evaluation involves the subjects’ assessment of their thoughts, desires, actions, and personality traits. For patients, self-evaluation mainly involves evaluating their decision-making and advocacy abilities. Items like “I am able to discuss tests and treatment options with my doctor” are used to evaluate this aspect. The items were scored on a 5-point Likert scale from 1 = “Strongly disagree” to 5 = “Strongly agree.” Higher ratings indicated higher levels of self-efficacy in decision-making. The Cronbach’s α of this scale was 0.851, the CR was 0.855, and the factor loadings ranged from 0.571 and 0.707. The AVE was 0.425, and the correlation coefficients between each item and the total score ranged from 0.645 to 0.756 (*p* < 0.01), demonstrating that the scale had good reliability and validity.

#### 3.2.3 Inpatient trust in physicians scale

This scale is based on the work of Chinese scholars Hongxia et al. (2015) and has been adjusted to fit the particularities of China’s medical service industry for this study. It includes four items, such as “I trust that the doctor comprehends the source of my illness.” Respondents rated their level of agreement on a 5-point Likert scale, from 1 = “Strongly disagree” to 5 = “Strongly agree.” Higher ratings indicated a greater level of trust in physicians. The Cronbach’s α was 0.922, CR was 0.923, and the factor loadings ranged between 0.828 and 0.906. The AVE was 0.751 and the correlation coefficients between each item and the total score ranged between 0.884 and 0.921 (*p* < 0.01), indicating good reliability and validity.

#### 3.2.4 Inpatient participation in medical decision-making scale

A self-developed three-item questionnaire was used. The items listed include the following: “I discussed disease examination, diagnosis, treatment, and other plans with the doctor,” “I discussed the final treatment plan with the doctor,” and “I demonstrated good compliance in participating in the medical decision-making process.” The questionnaire was scored on a 5-point Likert scale from 1 = “Strongly disagree” to 5 = “Strongly agree.” Higher ratings indicated greater levels of patient participation in decision-making. Cronbach’s α was 0.862, CR was 0.869, and the factor loadings ranged from 0.664 to 0.915. The AVE was 0.692 and the correlation coefficients between each item and the total score ranged from 0.808 to 0.924 (*p* < 0.01), indicating good reliability and validity.

#### 3.2.5 Inpatient satisfaction with medical services scale

This scale, adapted from Zhiwei and Jinghui (2020), comprised eight items, such as “I am pleased with the doctor’s medical skills.” Participants scored these items on a 5-point Likert scale ranging from 1 = “Strongly disagree” to 5 = “Strongly agree.” Higher ratings indicated higher levels of inpatient satisfaction with medical services. Cronbach’s α was 0.910, CR was 0.916, and the factor loadings ranged from 0.657 to 0.874. The AVE was 0.611 and the correlation coefficients between each item and the total score ranged from 0.747 to 0.851 (*p* < 0.01), indicating good reliability and validity.

### 3.3 Statistical analysis

Outliers and multicollinearity were evaluated in the preliminary analysis. Cook’s distance was used to detect outliers, considering a point as an outlier if the observed distance exceeded 0.5. The results indicated that the maximum Cook’s distance was 0.166, suggesting the absence of outliers. Multicollinearity was assessed using the variance inflation factor; all independent variables were less than 2, indicating the absence of multicollinearity.

Self-reported data were utilized in this study, which could potentially result in common method bias (Binbin et al., 2019). To assess this bias, Harman’s single-factor test was conducted on the collected data. The unrotated exploratory factor analysis revealed five factors with eigenvalues above 1, and the maximum factor variance explained was 39.84%, which is below 40%. As a result, it was determined that there is no significant common method bias present in this study.

SPSS 25.0 was used to conduct the statistical analysis. Means and standard deviations were utilized to describe quantitative variables, while frequencies and percentages were used to describe qualitative variables. Relationships among the variables were explored using Pearson’s correlation analysis. To analyze variations in inpatient satisfaction scores across demographic variables, the t-test was used to compare differences between two independent groups. One-way analysis of variance (ANOVA) was used to handle comparisons between multiple groups. Finally, a hierarchical multiple regression analysis was conducted to determine the key variables affecting inpatient satisfaction.

A structural equation model (SEM) was developed using AMOS 25.0 to study the specific mechanisms (direct or indirect, and their effect sizes) through which inpatient trust in physicians, self-efficacy, and participation in medical decision-making affected satisfaction with medical services. The model included two paths: trust in physicians → participation in decision making → satisfaction with medical service; and self-efficacy → participation in decision making → satisfaction with medical service. The mediating effect of inpatient participation in medical decision-making was examined using a bootstrap analysis, with a sampling number of 2,000. The model fit was evaluated using indicators such as χ^2^/df, RMSEA, GFI, AGFI, NFI, CFI, and TLI. The basic model was adjusted based on model modification indices to best fit the sample data.

## 4 Results

### 4.1 Correlation analysis of inpatient self-efficacy, trust in physicians, participation in medical decision-making, and satisfaction with medical services

The results indicated that patients scored highest on trust in physicians (*M* = 4.47, SD = 0.61) and lowest on self-efficacy (*M* = 3.63, SD = 0.77) among the four variables. There were significant correlations among the four variables: Patients’ self-efficacy (*r* = 0.319), trust in physicians (*r* = 0.714), and participation in medical decision-making (*r* = 0.565) positively correlated with their medical service satisfaction (*p* < 0.01). Further details are presented in Table 1.

**Table 1:** Correlation analysis of the variables.

Variable	Mean ± SD	1	2	3	4
1 Self-efficacy					
	3.63 ± 0.77	1			
2 Trust in physicians					
	4.47 ± 0.61	0.305**	1		
3 Participation in medical decision-making					
	4.10 ± 0.81	0.296**	0.488**	1	
4 Satisfaction with medical services					
	4.28 ± 0.63	0.319**	0.714**	0.565**	1

*P < 0.05, **P < 0.01; SD, standard deviation.

### 4.2 Stratified linear regression analysis of inpatient satisfaction with medical services

First, we assigned dummy variables to the nonordinal categorical data with statistical significance (*p* < 0.05) in the single-factor analysis of satisfaction with medical services for inpatients, such as household registration, hospital department, and decision-making method. Comparing the changes in △*R^2^*, trust in physicians had a greater impact on satisfaction with medical services than self-efficacy and participation in medical decision-making, explaining 36.7% of the variance (Supplementary Table S3). A comparison of the independent variables in Model 4 showed that inpatients’ satisfaction with medical services when decisions were made jointly by physicians and family members was higher than that of the reference group of inpatients where decisions were made independently by physicians (*β* = 0.080, *p* < 0.01). Trust in physicians had a significant positive impact on satisfaction with medical services (*β* = 0.553, *p* < 0.001), indicating that the higher patients’ level of trust in physicians, the higher their satisfaction (supporting H1). The self-efficacy of inpatients had a significant positive impact on their satisfaction with medical services (*β* = 0.069, *p* < 0.01), indicating that the higher the self-efficacy, the higher inpatients’ satisfaction with medical services (supporting H2). Participation in medical decision-making had a significant positive impact on satisfaction with medical services (*β* = 0.260, *p* < 0.001), indicating that the higher participants’ level of participation in medical decision-making, the higher their satisfaction (supporting H3).

### 4.3 Analysis of the mechanism of inpatient satisfaction with medical services

#### 4.3.1 Model construction

**Figure 2:**
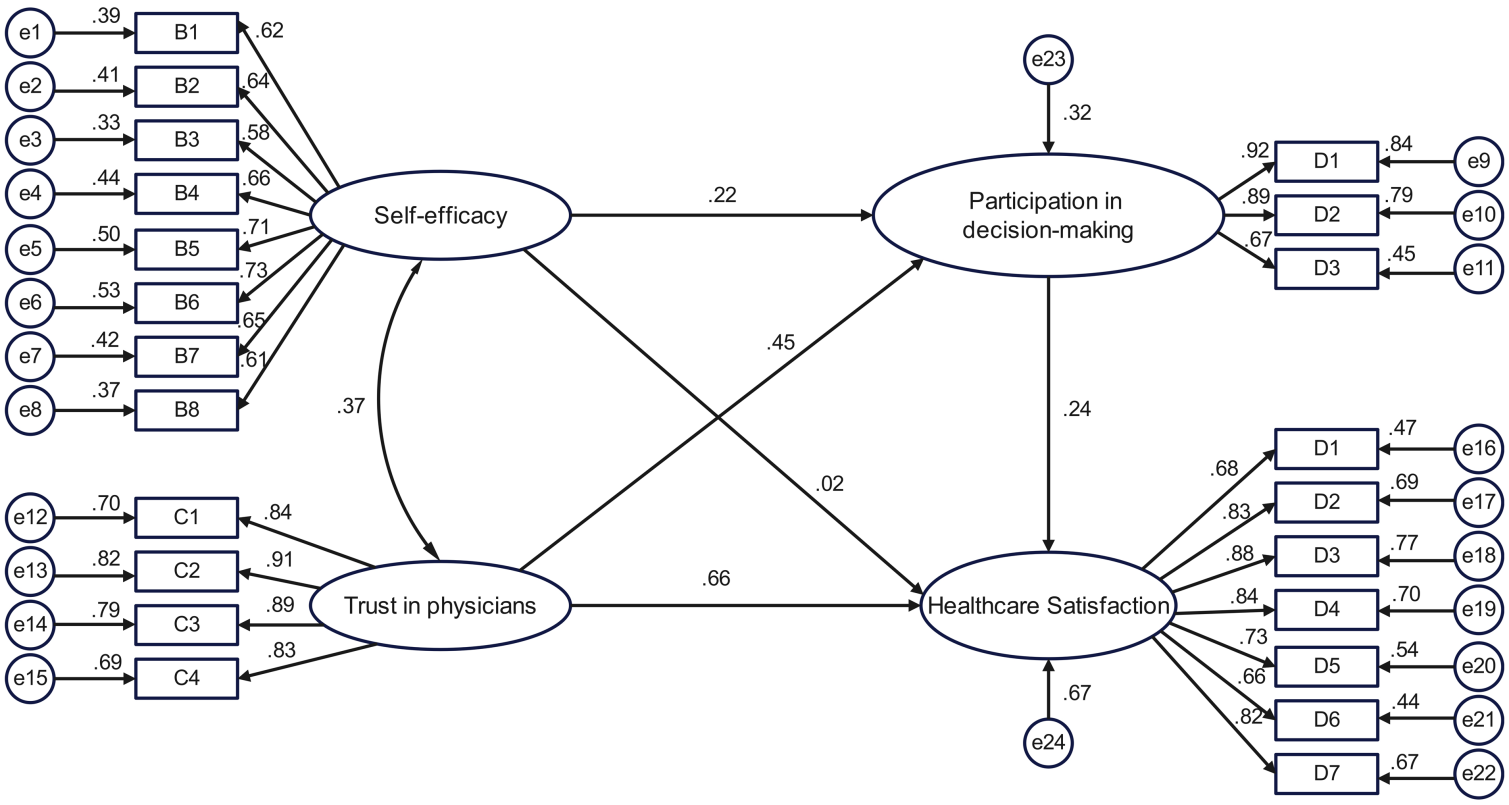
Initial model of the mechanism of inpatient satisfaction with medical services.

**Figure 3:**
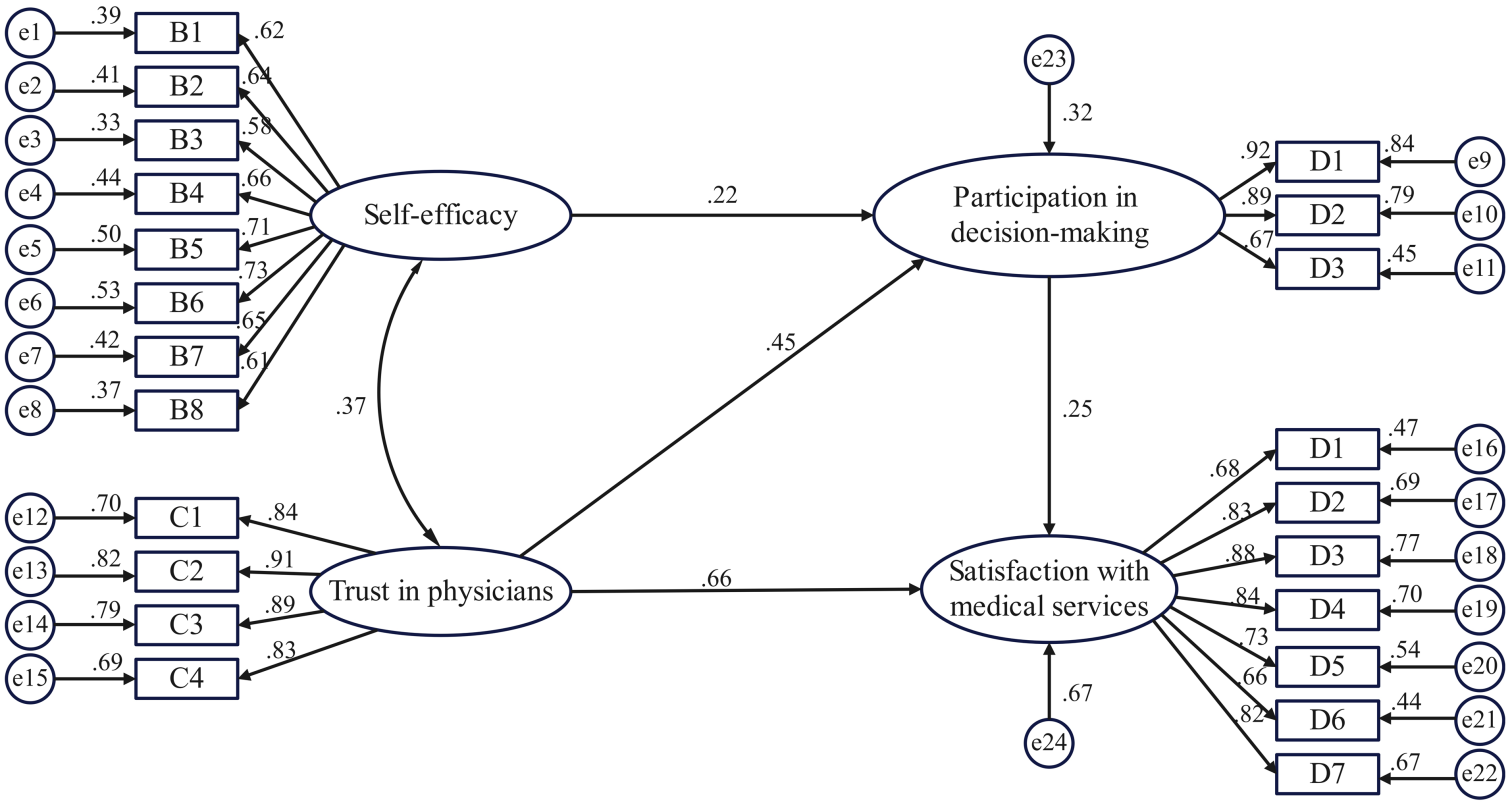
Modified model of the mechanism of inpatient satisfaction with medical services.

An SEM of inpatients’ satisfaction with medical services was constructed, with trust in physicians and self-efficacy as exogenous latent variables, participation in medical decision-making and satisfaction with medical services as endogenous latent variables, and specific questionnaire items as observed variables (Figure 2). The fit index of the initial model did not reach the desired value. Previous research (Bollen and Stine, 1992; Enders and An, 2005) suggests that the poor overall fit of the model may have been caused by the large sample size or the model itself. Based on this, we used the Bollen–Stine *p*-value correction (Bollen and Stine, 1992) to evaluate the model. In addition, the path from self-efficacy to satisfaction with medical services was not statistically significant (*p* >0 .05). Therefore, this path was excluded (Figure 3). After 2,000 rounds of bootstrap sampling correction, the *p*-value was <0.001, χ^2^/*df* (1.46) was in the range (Qian et al., 2015; Ling and Qun, 2014) of GFI, AGFI, NFI, CFI, TLI at >0.9, and RMSEA was less than 0.08, indicating that the SEM of inpatient satisfaction with medical services had a good overall fit (Table 2).

**Table 2:** Fitting results of the structural equation model.

Fit indices	Standards of fit indices	Original model	B-S modified model
*χ^2^*/*df*			
	1 < *χ2*/*df <* 3 good	10.40	1.46
RMSEA			
	<0.08 acceptable	0.11	0.02
GFI			
	>0.80 acceptable, >0.90 good	0.80	0.87
AGFI			
	>0.80 acceptable, >0.90 good	0.75	0.84
NFI			
	>0.80 acceptable, >0.90 good	0.83	0.86
CFI			
	>0.80 acceptable, >0.90 good	0.84	0.99
TLI			
	>0.80 acceptable, >0.90 good	0.82	0.99

#### 4.3.2 Path analysis of inpatient satisfaction with medical services

The results showed that trust in physicians and participation in medical decision-making positively influenced patient satisfaction with medical services, with standardized path coefficients of 0.661 and 0.248, respectively (*p* < 0.001). Thus, Hypotheses H1 and H3 were supported. In addition, trust in physicians had a direct impact on satisfaction with medical services and an indirect impact through the mediating variable of participation in decision-making, with an indirect effect of 0.110 and a mediating effect ratio of 14.3%. Hypothesis H4a was, thus, supported. Participation in medical decision-making played a mediating role between self-efficacy and patient satisfaction, with an effect value of 0.055, supporting H4b (Table 3).

**Table 3:** Path coefficients of inpatient satisfaction with medical services and hypotheses verification.

Relationships between variables	Standardized direct effect	Standardized indirect effect	Standardized total effect	*P-value*	Hypothesis supported/not supported
Trust in physicians ® Satisfaction with medical services					
	0.661	0.110	0.771	<0.001	H1 was supported
Self-efficacy ® Satisfaction with medical services					
	/	0.055	0.055	<0.001	H2 was not supported
Participation in medical decision-making ® Satisfaction with medical services					
	0.248	/	0.248	<0.001	H3 was supported

### 4.4 Mediating effect of participation in medical decision-making

The direct effect of patients’ trust in physicians on satisfaction with medical services was 0.661, and the indirect effect was 0.110. The 95% confidence interval did not contain zero, indicating a partial effect. Self-efficacy had no direct effect on satisfaction with medical services, with an indirect effect of 0.055. The 95% confidence interval did not contain zero, indicating a complete mediating effect (Supplementary Table S4).

## 5 Discussion

This study revealed the important roles of trust in physicians, self-efficacy, and participation in medical decision-making in hospitalized patients’ satisfaction with medical services. Specifically, participation in medical decision-making had a mediating effect on both trust in physicians and satisfaction with medical services, as well as between self-efficacy and satisfaction with medical services.

### 5.1 Impact of inpatient physician trust on satisfaction with medical services

Trust in physicians had a positive impact on inpatient satisfaction with medical services (supporting H1), which was consistent with the findings of Tang et al. (2019) and Zhang et al. (2020). This may be because trust plays a crucial role in the doctor–patient relationship. This view was also confirmed by Jneid et al. (2018), who found that when patients trust healthcare institutions and medical staff, their treatment compliance and treatment effect increase significantly (Yuan-Yuan et al., 2022), improving their satisfaction. Further, Chen et al. (2020) observed that when patients trusted their physicians, they were more willing to communicate any problems encountered during the medical process. Motivated by relationships built on trust, physicians addressed shortcomings in the medical process, thereby enhancing overall healthcare quality and service levels. Therefore, healthcare professionals should establish and maintain good doctor–patient relationships by actively listening to patient feedback, respecting patient needs, and establishing effective communication (Qi et al., 2023). In this way, they can enhance patient trust in physicians and the healthcare team, ultimately improving satisfaction.

### 5.2 Impact of inpatient self-efficacy on satisfaction with medical services

Self-efficacy was shown to have a positive impact on inpatients’ satisfaction with medical services (supporting H2). This result was consistent with that of Xian et al. (2023), who showed that patients with high self-efficacy had higher health literacy, better self-management and self-care abilities, and fewer difficulties in related care activities (Lee and Lin, 2011). This enhanced their confidence and ability to solve problems, thereby improving their satisfaction with medical services. Chang et al. (2019) also demonstrated that patients with high self-efficacy had higher satisfaction, possibly because they actively searched for relevant information about their illness and treatment and were more likely to understand and accept the information provided by physicians (Xunli, 2020), which could increase their satisfaction with medical services. Additionally, Zimmer et al. (2015) found that when patients were well-informed about a disease, they could anticipate treatment outcomes, experience less pain, stabilize their emotions (Yuanlin, 2018), and adapt better to disease treatment (Nielsen et al., 2013; Hirai et al., 2002), thus having a better healthcare experience. However, our finding was inconsistent with Wayment et al. (2020) study on patients in the emergency department. They found that even if patients had high self-efficacy and a good understanding of their illness, it was difficult for them to access health information or search for information related to their illness during emergencies, resulting in lower satisfaction. Therefore, healthcare providers should consider the importance of patient self-efficacy when providing medical services. Health education, special lectures, and case-sharing activities could help patients enhance their understanding of diseases and preventive care, gain alternative experiences by observing the behavior and results of other patients (Yun et al., 2015), and improve their recovery, confidence, and self-efficacy.

### 5.3 Impact of inpatient participation in medical decision-making on satisfaction with medical services

Participation in medical decision-making positively influenced satisfaction with medical services (supporting H3). This finding corresponds with that of Xiaoli et al. (2022). When patients participate in medical decision-making, they may develop a sense of responsibility and actively communicate with physicians. This enables them to better understand various treatment options (Yanhua, 2021), allowing them to choose the most suitable option for their needs and, ultimately, enhancing their satisfaction. Chen et al. (2019) also found that participating in medical decision-making could increase patients’ willingness to seek medical treatment and ensure their medical autonomy, leading to an overall higher level of satisfaction with medical services. Furthermore, Shukui (2019) showed that patients who participated in the decision-making process felt respected and cared for by their physicians, which increased their trust in their physicians and resulted in higher levels of satisfaction. Patient participation in medical decision-making has, consequently, become a trend in global healthcare (Shay and Lafata, 2015). Healthcare professionals can provide medical information through multiple channels to help patients actively participate in medical decision-making (Cai et al., 2021). It is also important for healthcare providers to understand patients’ decision-making preferences in advance and encourage active participation in medical decision-making. Additionally, hospitals can incorporate communication and expression training into routine physician training programs (Yijun and Meiqiong, 2013) to promote patient participation.

### 5.4 Mediating effect of inpatient participation in medical decision-making

Bootstrap tests revealed that patients’ participation in medical decision-making during their inpatient stay partially mediated the link between their trust in physicians and satisfaction with medical services. This finding supports hypothesis H4a. A portion of the effect of patients’ trust in physicians on their satisfaction with medical services was achieved through the indirect effect of their participation in medical decision-making. This finding is similar to that of Jiejing and Bin (2010), who observed that when patients trusted their physicians, they raised doubts (Peek et al., 2009), cooperated with physicians during diagnosis and treatment, and participated in medical decision-making, leading to favorable medical outcomes and, ultimately, enhancing patient satisfaction. This finding also aligns with the SDT (Deci and Ryan, 1985), which suggests that patients’ trust in physicians satisfies their psychological relationship needs, enhances their intrinsic motivation, and stimulates their participation in medical decision-making, thereby improving their healthcare experience. However, this finding contradicts the results of a study by Pokhilenko et al. (2021), which showed that a high level of trust impedes patients’ participation in medical decision-making. This discrepancy may be specific to the good doctor–patient relationships in the Netherlands, where patients generally have a high level of trust in physicians and believe that physicians act in their best interests, thus relying more on the advice given by physicians and reducing their subjective agency. As such, physicians should focus on building good interpersonal relationships when interacting with patients, as well as promoting information exchange and patient participation in decision-making by increasing trust and improving patient satisfaction with medical services.

Bootstrap tests revealed that inpatient participation in medical decision-making fully mediated the relationship between patients’ self-efficacy and their satisfaction with medical services (supporting H4b). This finding was consistent with that of Xin and Cailan (2021). Patients with high self-efficacy can better understand physicians’ explanations of their illnesses and can fully utilize their initiative and agency. This not only gives them a greater sense of participation but also improves the quality of medical decision-making, ultimately enhancing patients’ satisfaction with the healthcare experience. This finding also aligns with the self-efficacy theory, which suggests that individuals’ self-efficacy can influence their satisfaction by regulating their participation in medical decision-making (Xiaolian et al., 2004). Therefore, hospitals can improve patient self-efficacy through health education, which can influence patients’ participation in medical decision-making and ultimately improve satisfaction.

### 5.5 Implications and limitations

This study utilized SEM to expand the existing literature on inpatients’ satisfaction with medical services. The study clarified the mechanism of influence between patient trust in physicians, self-efficacy, patient participation in medical decision-making, and satisfaction with medical services, enriching the application of self-efficacy theory in the healthcare field. Additionally, it proposed measures to promote patient trust in physicians, self-efficacy, and participation in medical decision-making, providing a reference for healthcare institutions to improve patient satisfaction.

However, this study has three major limitations. First, as a cross-sectional study, it could only demonstrate correlations between research variables and could not establish causality. To confirm causal conclusions, future research could consider using a two-time point or longitudinal cross-sectional study. Second, we only included inpatients from secondary and tertiary public hospitals in Hangzhou. The generalizability of the findings may, thus, be limited because Hangzhou has a thriving economy. Therefore, future research should include underdeveloped areas, such as those in central and western China, in the survey scope. Third, this study relied on a questionnaire survey to collect data from hospitalized patients, and the outcomes were self-reported. This method may have introduced reporting bias. As such, future research could also collect evaluations from patients’ family members, healthcare personnel, and other actors to address this bias.

## 6 Conclusion

This study indicated that inpatient’s trust in physicians and self-efficacy directly influenced their satisfaction with medical services and could also indirectly influence satisfaction through participation in medical decision-making. Consequently, healthcare institutions should provide training on service attitudes for medical personnel, establish patient-oriented service concepts, enhance communication methods and skills, and strengthen patient trust. Moreover, healthcare institutions should regularly offer health education for inpatients to enhance their disease awareness, build their confidence in recovery, and improve patient self-efficacy. To promote patients’ participation and sense of responsibility for their own health, healthcare providers could encourage them to engage in medical decision-making, which could increase their satisfaction with medical services.

## Data Availability

The raw data supporting the conclusions of this article will be made available by the authors, without undue reservation.

## References

[ref1] Alsarhan M. A. Alaqeely R. S. Aljasser R. Otaibi D. H. Aloraini S. Alshiddi I. F. (2021). Evaluation of complacency about dental implants with shared decision making and satisfaction scores: a cross-sectional study. Saudi. Dent. J. 33, 929–936. doi: 10.1016/j.sdentj.2021.09.001, PMID: 34938034 PMC8665187

[ref2] Arnetz J. E. Almin I. Bergström K. Franzén Y. Nilsson H. (2004). Active patient involvement in the establishment of physical therapy goals: effects on treatment outcome and quality of care. Adv. Physiother. 6, 50–69. doi: 10.1080/14038190310017147

[ref3] Audrain-Pontevia A. F. Menvielle L. (2018). Effects of interpersonal trust among users of online health communities on patient trust in and satisfaction with their physician. Int. J. Technol. Assess. Health Care 34, 56–62. doi: 10.1017/S0266462317004433, PMID: 29427998

[ref4] Baiardini I. Rogliani P. Santus P. Corsico A. G. Contoli M. Scichilone N.. (2019). Disease awareness in patients with COPD: measurement and extent. Int. J. Chron. Obstruct. Pulm. Dis. 14, 1–11. doi: 10.2147/COPD.S179784, PMID: 30587957 PMC6301728

[ref5] Binbin H. Shengqi Z. Xinchun W. Liu C. (2019). The association between father coparenting behavior and adolescents’ peer attachment: the mediating role of father-child attachment and the moderating role of adolescents’ neuroticism. Psychol. Dev. Educ. 35, 176–183. doi: 10.16187/j.cnki.issn1001-4918.2019.02.06

[ref6] Birkhäuer J. Gaab J. Kossowsky J. Hasler S. Krummenacher P. Werner C.. (2017). Trust in the health care professional and health outcome: a meta-analysis. PLoS One 12:e0170988. doi: 10.1371/journal.pone.0170988, PMID: 28170443 PMC5295692

[ref7] Bollen K. A. Stine R. A. (1992). Bootstrapping goodness-of-fit measures in structural equation models. Sociol. Methods Res. 21, 205–229. doi: 10.1177/0049124192021002004

[ref8] Buttitta M. Rousseau A. Guerrien A. (2017). A new understanding of quality of life in children and adolescents with obesity: contribution of the self-determination theory. Curr. Obes. Rep. 6, 432–437. doi: 10.1007/s13679-017-0281-8, PMID: 29052152

[ref9] Cai J. Xiangyu L. Xiaowei P. Ouying C. Shuangshuang L. Wenjing H.. (2021). Satisfaction of patients participating in medical decision-making after oral thyroidectomy and its correlation with doctor–patient relationship. J. Nurs. Train 36, 476–479. doi: 10.16821/j.cnki.hsjx.2021.05.020

[ref10] Centre for Health Statistics and information (2021). The sixth national health services statistics survey report (2018). Beijing: People’s Medical Publishing House.

[ref11] Chang Z. E. Zheng H. L. Zhu Hui Y. Song B. (2019). Study on the influence mechanism of the use of hospital intelligent medical system on patients’ satisfaction: based on the perspective of technology acceptance model. Chin. Hosp. Manag. 39, 61–64.

[ref12] Chen W. Feng Y. Fang J. Wu J. Huang X. Wang X.. (2020). Effect of trust in primary care physicians on patient satisfaction: a cross-sectional study among patients with hypertension in rural China. BMC Fam. Pract. 21:196. doi: 10.1186/s12875-020-01268-w, PMID: 32957936 PMC7507258

[ref13] Chen T. R. Huangl Y. Lifang C. Lu P. (2019). Influence of shared-decision making mode on harmonious doctor–patient relationship under new social contradictions. China Med. 27, 48–51. doi: 10.19338/j.issn.1672-2019.2019.10.013

[ref14] Chughtai A. A. Buckley F. (2011). Work engagement: antecedents, the mediating role of learning goal orientation and job performance. Career Dev. Int. 16, 684–705. doi: 10.1108/13620431111187290

[ref15] Deci E. L. Ryan R. M. (1985). Intrinsic motivation and self-determination in human behavior. New York: Springer.

[ref16] Denny M. C. Vahidy F. Vu K. Y. T. Sharrief A. Z. Savitz S. I. (2017). Video-based educational intervention associated with improved stroke literacy, self-efficacy, and patient satisfaction. PLoS One 12:e0171952. doi: 10.1371/journal.pone.0171952, PMID: 28333925 PMC5364024

[ref17] Enders C. K. An S. A. S. (2005). Macro for implementing the modified bollen-Stine bootstrap for missing data: implementing the bootstrap using existing structural equation modeling software. Struct. Equ. Model. Multidiscip. J. 12, 620–641. doi: 10.1207/s15328007sem1204_6

[ref18] Giesler J. M. Weis J. (2008). Developing a self-rating measure of patient competence in the context of oncology: a multi-center study. Psycho-Oncology 17, 1089–1099. doi: 10.1002/pon.1330, PMID: 18318001

[ref19] Haijun X. (2012). A study on the relationship between hospitalization information acquisition and patient satisfaction in patients with acute myocardial infarction. Mod. Med. J. China 14, 88–90. doi: 10.3969/j.issn.1672-9463.2012.12.034

[ref20] Hangzhou Municipal Bureau of Statistics, Hangzhou Socio-economic Survey Team. (2022). Available at: http://tjj.hangzhou.gov.cn/art/2023/3/20/art_1229279682_4149703.html?eqid=81961fdd003cbadf000000026443c3ae (Accessed August 27, 2023)

[ref21] Hirai K. Suzuki Y. Tsuneto S. Ikenaga M. Hosaka T. Kashiwagi T. (2002). A structural model of the relationships among self-efficacy, psychological adjustment, and physical condition in Japanese advanced cancer patients. Psycho-oncol 11, 221–229. doi: 10.1002/pon.561, PMID: 12112482

[ref22] Hongxia Z. Xinhai W. Baogang Z. (2015). Relationship among interaction, presence and consumer trust in B2C online shopping. Manag. Rev. 27, 43–54. doi: 10.14120/j.cnki.cn11-5057/f.2015.02.005

[ref23] Hood C. Patton R. (2022). Exploring the role of psychological need fulfilment on stress, job satisfaction and turnover intention in support staff working in inpatient mental health hospitals in the NHS: a self-determination theory perspective. J. Ment. Health 31, 692–698. doi: 10.1080/09638237.2021.1979487, PMID: 34565267

[ref24] Hu L. Ding H. Liu S. Wang Z. Hu G. Liu Y. (2020). Influence of patient and hospital characteristics on inpatient satisfaction in China's tertiary hospitals: a cross-sectional study. Health Expect. 23, 115–124. doi: 10.1111/hex.12974, PMID: 31637800 PMC6978851

[ref25] Jiahui W. Kai L. Aiping W. (2019). The application and effect evaluation of self-efficacy theory-based intervention for self-management behavior modification in patients with enterostomy. J. China Med. Univ. 48, 1041–1044. doi: 10.12007/j.issn.0258-4646.2019.11.019

[ref26] Jiangyan X. Jian M. I. N. Daimin L. (2023). Relationship between shared decision-making and patients’ satisfaction in outpatient department of obstetrics and gynecology. Mod. Hosp. 23:695. doi: 10.3969/j.issn.1671i.org/10.3969/j.i

[ref27] Jiejing Y. Bin Z. (2010). Research and evaluation of interpersonal citizenship behavior and its antecedents and outcome variables. Manag. Rev. 22, 69–75. doi: 10.14120/j.cnki.cn11-5057/f.2010.05.010

[ref28] Jneid S. Jabbour H. Hajj A. Sarkis A. Licha H. Hallit S.. (2018). Quality of life and its association with treatment satisfaction, adherence to medication, and trust in physician among patients with hypertension: a cross-sectional designed study. J. Cardiovasc. Pharmacol. Ther. 23, 532–542. doi: 10.1177/1074248418784292, PMID: 29916266

[ref29] Lee Y. Y. Lin J. L. (2011). How much does trust really matter? A study of the longitudinal effects of trust and decision-making preferences on diabetic patient outcomes. Patient Educ. Couns. 85, 406–412. doi: 10.1016/j.pec.2010.12.005, PMID: 21269794

[ref30] Ling X. Qun M. (2014). One of the results of the fifth National Health Service survey – resident satisfaction. Chin. J. Health Inform. Manag. 11, 104–105. doi: 10.3969/j.issn.1672-5166.2014.02.01

[ref31] Min C. Yao-guang Z. (2010). Inpatients’ evaluation of the health institution and the factors associated with patients’ dissatisfaction. Chin. Hosp. Manag. 30, 7–9. doi: 10.3969/j.issn.1001-5329.2010.05.003

[ref32] Ming Y. Qiansha W. Rongrong H. Yunyun J. Yaling L. Cai P.. (2024). Study on factors influencing alarm fatigue among ICU nurses. J. Nurs. Sci. 39, 27–31. doi: 10.3870/j.issn.1001-4152.2024.06.027

[ref33] Molina Y. Kim S. Berrios N. Calhoun E. A. (2015). Medical mistrust and patient satisfaction with mammography: the mediating effects of perceived self-efficacy among navigated African American women. Health Expect. 18, 2941–2950. doi: 10.1111/hex.12278, PMID: 25308749 PMC4393336

[ref34] Nakayama K. Osaka W. Matsubara N. Takeuchi T. Toyoda M. Ohtake N.. (2020). Shared decision making, physicians’ explanations, and treatment satisfaction: a cross-sectional survey of prostate cancer patients. BMC Med. Inform. Decis. Mak. 20:334. doi: 10.1186/s12911-020-01355-z, PMID: 33317523 PMC7734751

[ref35] Nielsen B. K. Mehlsen M. Jensen A. B. Zachariae R. (2013). Cancer-related self-efficacy following a consultation with an oncologist. Psycho-Oncology 22, 2095–2101. doi: 10.1002/pon.3261, PMID: 23463726

[ref36] Obeidat R. F. Lally R. M. (2018). Jordanian physicians’ perceived barriers and facilitators to patient participation in treatment decision-making: an exploratory study. Indian J. Cancer 55, 377–381. doi: 10.4103/ijc.IJC_122_18, PMID: 30829274

[ref37] Peek M. E. Wilson S. C. Gorawara-Bhat R. Odoms-Young A. Quinn M. T. Chin M. H. (2009). Barriers and facilitators to shared decision-making among African-Americans with diabetes. J. Gen. Intern. Med. 24, 1135–1139. doi: 10.1007/s11606-009-1047-0, PMID: 19578818 PMC2762499

[ref38] Pokhilenko I. van Esch T. E. M. Brabers A. E. M. de Jong J. D. (2021). Relationship between trust and patient involvement in medical decision-making: a cross-sectional study. PLoS One 16:e0256698. doi: 10.1371/journal.pone.0256698, PMID: 34437626 PMC8389380

[ref39] Qi C. Chi Z. Fang T. Sihong L. (2023). Impact of doctor–patient relationship on grass-roots health management services in Hangzhou based on trust perspective. Med. Soc. 36:65. doi: 10.13723/j.yxysh.2023.03.010

[ref40] Qian Y. Xiaohe W. Yajing C. Shuangzhu Z. Caiyong Y. Gu Y. (2015). Studying on the progress of research in patients’ satisfaction on medical services and its problems. Chin. Health Serv. Manag. 32, 105–107.

[ref41] Shan L. Li Y. Ding D. Wu Q. Liu C. Jiao M.. (2016). Patient satisfaction with hospital inpatient care: effects of trust, medical insurance and perceived quality of care. PLoS One 11:e0164366. doi: 10.1371/journal.pone.0164366, PMID: 27755558 PMC5068749

[ref42] Shangxin C. (2022). Institutional or cultural: factors influencing patient trust in China and its evolution. J Sociol Stud 37:229.

[ref43] Shay L. A. Lafata J. E. (2015). Where is the evidence? A systematic review of shared decision making and patient outcomes. Med. Decis. Mak. 35, 114–131. doi: 10.1177/0272989X14551638 PMC4270851 25351843

[ref44] Shukui L. (2019). Value analysis of doctor–patient shared decision making in diagnosis and treatment of breast cancer. Med. Soc. 32, 61–63. doi: 10.13723/j.yxysh.2019.08.015

[ref45] Steca P. Greco A. Monzani D. Politi A. Gestra R. Ferrari G.. (2013). How does illness severity influence depression, health satisfaction and life satisfaction in patients with cardiovascular disease? The mediating role of illness perception and self-efficacy beliefs. Psychol. Health 28, 765–783. doi: 10.1080/08870446.2012.759223, PMID: 23343116

[ref46] Suh W. S. Lee C. K. (2010). Impact of shared-decision making on patient satisfaction. J. Prev. Med. Public Health 43, 26–34. doi: 10.3961/jpmph.2010.43.1.26 20185980

[ref47] Tan W. Zhang W. Li H. Tian D. Wang P. Deng X.. (2015). Analysis of satisfactions for services among outpatients and inpatients at the age of equal to or more than 15 years old in Hunan Province. J. Int. Soc. Sports Nutr. 40, 1148–1155. doi: 10.11817/j.issn.1672-7347.2015.10.016, PMID: 26541852

[ref48] Tang C. Tian B. Zhang X. Zhang K. Xiao X. Simoni J. M.. (2019). The influence of cultural competence of nurses on patient satisfaction and the mediating effect of patient trust. J. Adv. Nurs. 75, 749–759. doi: 10.1111/jan.13854, PMID: 30209816

[ref49] Tung Y. C. Chang G. M. (2009). Patient satisfaction with and recommendation of a primary care provider: associations of perceived quality and patient education. Int. J. Qual. Health Care 21, 206–213. doi: 10.1093/intqhc/mzp006, PMID: 19258342

[ref50] Waters S. Edmondston S. J. Yates P. J. Gucciardi D. F. (2016). Identification of factors influencing patient satisfaction with orthopaedic outpatient clinic consultation: a qualitative study. Man. Ther. 25, 48–55. doi: 10.1016/j.math.2016.05.334, PMID: 27422597

[ref51] Wayment A. Wong C. Byers S. Eley R. Boyde M. Ostini R. (2020). Beyond access block: understanding the role of health literacy and self-efficacy in low-acuity emergency department patients. Ochsner J. 20, 161–169. doi: 10.31486/toj.19.0047, PMID: 32612470 PMC7310186

[ref52] Xia L. Ma C. Ai Y. Zhao L. Tang X. Zhang X.. (2015). Analysis and evaluation of inpatients' satisfaction degree of 652 cases. Chin. Hosp. Manag. 35, 59–61.

[ref53] Xian C. Qingyun J. Shiya X. Chen T. Gong L. (2023). Analysis of the influence of self-efficacy intervention on the compliance and satisfaction of cancer patients during PICC catheterization. Chin. Med. Her. 20, 189–192. doi: 10.20047/j.issn1673-7210.2023.04.43

[ref54] Xiaohui Z. (2020) Study on inpatient satisfaction index model in public hospitals ─illustrated by the case of a hospital. Master’s thesis. Tianjin, Tianjin University.

[ref55] Xiaoli D. Gaiping X. U. Hongjian L. Lihong L. Huan Z. (2022). Correlation analysis of the level of participation in medical decision-making and satisfaction with medical service quality in patients with hearing loss. Mod. Med. Health 38:2939. doi: 10.3969/j.issn.1009-5519.2022.17.011

[ref56] Xiaolian J. Yonghong X. Hui L. I. U. (2004). Bandura self-efficacy theory and its implications for nursing education. J Nurs Train 4, 352–354. doi: 10.16821/j.cnki.hsjx.2004.04.036

[ref57] Xin W. Cailan Z. (2021). The relationship between self-efficacy and learning satisfaction in college students’ MOOC learning: a mediating effect analysis of learning participation. China Inf. Technol. Educ. 21, 105–109. doi: 10.3969/j.issn.1674-2117.2021.21.036

[ref58] Xue J. (2017) Revise and application of client empowerment scale in hospitalized patients with chronic diseases. Master’s thesis. Guangzhou, Southern Medical University.

[ref59] Xunli X. (2020) Study on the effect of patient health literacy on patient participation satisfaction under online consultation. Master’s thesis. Wuhan: Huazhong University of Science and Technology.

[ref60] Yan M. Zhi M. Xu Y. Hu L. Liu Y. (2022). Inpatient satisfaction with nursing care and its impact factors in Chinese tertiary hospitals: a cross-sectional study. Int. J. Environ. Res. Public Health 19:16523. doi: 10.3390/ijerph192416523, PMID: 36554403 PMC9778790

[ref61] Yanhua X. (2021) Study of the influence of shared decision-making on inpatient satisfaction. Master’s thesis. Guangzhou: Southern Medical University.

[ref62] Yijun Y. Meiqiong Y. (2013). Literature review on patients’ involvement in decision-making in medical therapy and nursing care: the influencing factors. J. Nurs. Sci. 28, 93–96. doi: 10.3870/hlxzz.2013.05.093

[ref63] Yingru X. (2021) Research on the driving factors and promoting strategies of inpatients’ participation in medical decision-making behavior. Master’s thesis. Hangzhou, Hangzhou Normal University.

[ref64] Yuanlin H. (2018). Research on the influencing factors of student learning satisfaction in local undergraduate colleges: based on the perspective of students’ self-learning effectiveness. Higher Educ. Explor. 3, 43–50. doi: 10.3969/j.issn.1673-9760.2018.03.008

[ref65] Yuan-Yuan D. Yue L. I. Lin M. A. Shuo Z. Rong T. (2022). Analysis of satisfaction and influencing factors of inpatients’ medical service. China J. Pharm. Econ. 17:62. doi: 10.12010/j.issn.1673-5846.2022.06.009

[ref66] Yun T. Ze-wen L. Ying-wen L. I. Jian-zhen L. A. N. Yu-mei C. (2015). Influence of self-efficacy training on nutrition of patients with maintenance hemodialysis. J. Nurs. 22, 47–49. doi: 10.16460/j.issn1008-9969.2015.20.047

[ref67] Zhang H. Zhang R. Lu X. Zhu X. (2020). Impact of personal trust tendency on patient compliance based on internet health information seeking. Telemed. J. E Health 26, 294–303. doi: 10.1089/tmj.2018.0296, PMID: 31045486

[ref68] Zhenhua J. Ling C. Yong L. (2023). Effect of self-efficacy-based intelligent walking training on function of lower extremities of stroke patients. Chin. J. Rehabil. Theor. Pract. 29, 504–509. doi: 10.3969/j.issn.1006-9771.2023.05.002

[ref69] Zhiwei Z. Jinghui Y. (2020). Analysis of influencing factors of inpatient satisfaction and policy implications: based on the survey of a public hospital in Xuzhou. J Jinzhou Univ (Soc Sci Ed) 18, 52–57. doi: 10.13847/j.cnki.lnmu(sse).2020.05.015

[ref70] Zimmer A. Bläuer C. Coslovsky M. Kappos L. Derfuss T. (2015). Optimizing treatment initiation: effects of a patient education program about fingolimod treatment on knowledge, self-efficacy and patient satisfaction. Mult. Scler. Relat. Disord. 4, 444–450. doi: 10.1016/j.msard.2015.06.010, PMID: 26346793

