# Supplementary material for: Impact of inpatient self-efficacy and trust in physicians on inpatient satisfaction with medical services: the mediating role of patient participation in medical decision-making
Source: Front Psychol. 2024 Aug 30;15:1364319. doi: 10.3389/fpsyg.2024.1364319 (PMC11392843; doi:10.3389/fpsyg.2024.1364319)
Supplement: Supplementary file 1 [file Data_Sheet_1.docx]

Supplementary Material

# Supplementary Tables

**Table S1.** *Participant characteristics (N = 814)*

| Characteristic | Frequency (N) | Composition ratio (%) |
| --- | --- | --- |
| **Sex** |  |  |
| Male | 404 | 49.6 |
| Female | 410 | 50.4 |
| **Age** |  |  |
| ≤25 | 77 | 9.5 |
| 26–35 | 204 | 25.1 |
| 36–45 | 186 | 22.9 |
| 46–55 | 165 | 20.3 |
| ≥56 | 182 | 22.4 |
| **Level of education** |  |  |
| Junior high school and below | 279 | 34.3 |
| High school/Technical secondary school | 188 | 23.1 |
| College | 145 | 17.8 |
| Undergraduate | 164 | 20.1 |
| Master’s degree or above | 38 | 4.7 |
| **Marital status** |  |  |
| Married | 684 | 84.0 |
| Unmarried | 107 | 13.1 |
| Other | 23 | 2.8 |
| **Household registration** |  |  |
| Rural | 404 | 49.6 |
| Urban | 410 | 50.4 |
| **Type of occupation** |  |  |
| Public service/Government employee | 83 | 10.2 |
| Company employee | 170 | 20.9 |
| Enterprise worker | 112 | 13.8 |
| Freelancer | 225 | 27.6 |
| Migrant worker | 42 | 5.2 |
| Farm worker | 47 | 5.8 |
| Retiree | 92 | 11.3 |
| Student | 32 | 3.9 |
| Other | 11 | 1.4 |
| **Monthly household income (RMB)** |  |  |
| ＜10,000 | 310 | 38.1 |
| 10000–20000 | 304 | 37.3 |
| 20001–30000 | 113 | 13.9 |
| ＞30,000 | 87 | 10.7 |
| **Type of health insurance** |  |  |
| None | 18 | 2.2 |
| Urban Employee Medical Insurance | 435 | 53.4 |
| Urban and Rural Resident Medical Insurance (including New Rural Cooperative Medical Insurance and Urban Resident Medical Insurance) | 279 | 34.3 |
| Commercial Medical Insurance | 14 | 1.7 |
| Urban Employee Medical Insurance and Urban and Rural Resident Medical Insurance | 11 | 1.4 |
| Urban Employee Medical Insurance and Commercial Medical Insurance | 41 | 5.0 |
| Urban and Rural Resident Medical Insurance and Commercial Medical Insurance | 16 | 2.0 |
| **Current hospital department** |  |  |
| Internal Medicine | 176 | 21.6 |
| Surgery | 378 | 46.4 |
| Gynecology | 86 | 10.6 |
| Otorhinolaryngology | 82 | 10.1 |
| Other | 92 | 11.3 |
| **Hospitalizations in the past year (times)** |  |  |
| 0 | 525 | 64.5 |
| 1 | 183 | 22.5 |
| 2 | 43 | 5.3 |
| ≥3 | 63 | 7.7 |
| **Current method of decision-making** |  |  |
| Doctor makes the decision independently | 165 | 20.3 |
| Patient or patient’s family make the decision independently | 29 | 3.6 |
| Doctor and patient make the decision together | 322 | 39.6 |
| Patient and family make the decision together | 26 | 3.2 |
| Doctor and family make the decision together | 131 | 16.1 |
| Doctor, patient, and family make the decision together | 141 | 17.3 |
| **Desired method of decision-making** |  |  |
| Doctor makes the decision independently | 28 | 3.4 |
| Patient or patient’s family make the decision independently | 7 | 0.9 |
| Doctor and patient make the decision together | 153 | 18.8 |
| Patient and family make the decision together | 16 | 2.0 |
| Doctor and family make the decision together | 112 | 13.8 |
| Doctor, patient, and family make the decision together | 498 | 61.2 |

**Table S2.** *Measurement items and results of reliability and validity analyses (N = 814)*

| Variable | Item | Load ^a^ | Cronbach′s α | Correlation coefficient | CR | AVE |
| --- | --- | --- | --- | --- | --- | --- |
| Self-efficacy | I can find information about diseases and treatments from books and the Internet. | 0.702 | 0.851 | 0.748** | 0.855 | 0.425 |
|  | I can find alternative treatment methods, such as acupuncture and massage. | 0.707 |  | 0.756** |  |  |
|  | I can proactively inform the doctor about my medical history, allergies, and symptoms. | 0.619 |  | 0.645** |  |  |
|  | I can express my needs and expectations to the doctor. | 0.675 |  | 0.693** |  |  |
|  | I have the ability to discuss examination and treatment plans with the doctor. | 0.648 |  | 0.712** |  |  |
|  | I have the ability to choose different treatment options (surgery, medication, etc.). | 0.668 |  | 0.734** |  |  |
|  | If I am unsatisfied during the treatment process, I will report it to the department head, hospital, or relevant department. | 0.615 |  | 0.680** |  |  |
|  | If there is a dispute, I know how to resolve it (who to report to, knowledge of complaint hotline, etc.). | 0.571 |  | 0.654** |  |  |
| Trust in physicians | I believe that the doctor has a clear understanding of my condition. | 0.841 | 0.922 | 0.886** | 0.923 | 0.751 |
|  | I have great confidence in the doctor’s work attitude. | 0.906 |  | 0.921** |  |  |
|  | I believe that the doctor is highly skilled in treatment techniques. | 0.888 |  | 0.913** |  |  |
|  | I believe that the doctor will prioritize my health. | 0.828 |  | 0.884** |  |  |
| Participation in medical decision-making | I discuss disease examination, diagnosis, and treatment plans with the doctor. | 0.915 | 0.862 | 0.924** | 0.869 | 0.692 |
|  | The doctor and I jointly decide on the final treatment plan. | 0.894 |  | 0.919** |  |  |
|  | I show good compliance in participating in the medical decision-making process. | 0.664 |  | 0.808** |  |  |
| Satisfaction with medical services | I am happy with the ward environment and facilities (such as beds, bedding, etc.). | 0.689 | 0.910 | 0.765** | 0.916 | 0.611 |
|  | I am happy with the doctor’s explanations and treatment of my condition. | 0.830 |  | 0.851** |  |  |
|  | I am happy with the doctor’s medical skills. | 0.874 |  | 0.857** |  |  |
|  | I am happy with the communication and interaction with the doctor. | 0.837 |  | 0.831** |  |  |
|  | I feel that my condition has improved. | 0.736 |  | 0.790** |  |  |
|  | I believe that the medical expenses for this hospitalization are reasonable. | 0.657 |  | 0.747** |  |  |
|  | Overall, I am satisfied with the medical service provided this time. | 0.822 |  | 0.851** |  |  |

*Note.* **At the 0.01 level (double-tailed), the correlation is significant. CR, composite reliability; AVE, average variance extracted

**Table S3.** *Stratified regression analysis of inpatient satisfaction with medical services*

| Variable | First block | Second block | Third block | Fourth block |
| --- | --- | --- | --- | --- |
|  | Standardized *Beta* | Standardized *Beta* | Standardized *Beta* | Standardized *Beta* |
| Household registration (rural = reference group) |  |  |  |  |
| Urban | -0.061 | -0.089** | -0.021 | -0.023 |
| Current hospital department (Internal Medicine = reference group) |  |  |  |  |
| Surgery | 0.150** | 0.158*** | 0.076* | 0.014 |
| Gynecology | 0.013 | -0.005 | 0.016 | -0.006 |
| Otorhinolaryngology | 0.076 | 0.054 | 0.034 | 0.021 |
| Other | 0.099* | 0.070 | 0.052 | 0.031 |
| Method of decision-making for this plan (Doctor makes the decision independently = reference group) |  |  |  |  |
| Patient or patient’s family makes the decision independently | 0.059 | 0.047 | 0.062* | 0.058* |
| Doctor and patient make the decision together | 0.192*** | 0.131** | 0.089** | 0.067* |
| Patient and family make the decision together | -0.026 | -0.031 | 0.001 | 0.001 |
| Doctor and family make the decision together | 0.192*** | 0.178*** | 0.108*** | 0.080** |
| Doctor, patient, and family make the decision together | 0.213*** | 0.147*** | 0.079*** | 0.051 |
| Self-efficacy |  | 0.321*** | 0.114*** | 0.069** |
| Trust in physicians |  |  | 0.656*** | 0.553*** |
| Participation in medical decision-making |  |  |  | 0.260*** |
| *R^2^* | 0.074 | 0.170 | 0.537 | 0.583 |
| *F* | 6.387*** | 14.906*** | 77.336*** | 86.128*** |
| △*R^2^* | 0.074 | 0.096 | 0.367 | 0.047 |
| △*F* | 6.387*** | 92.790*** | 634.544*** | 89.314*** |
| VIF_max_ | 1.794 | 1.833 | 1.838 | 1.848 |

*Note.* The independent variables are divided into four blocks: Model 1: demographic variables, Model 2: demographic variables + self-efficacy, Model 3: demographic variables + self-efficacy + trust in physicians, Model 4: demographic variables + self-efficacy + trust in physicians + participation in medical decision-making. When demographic characteristics, self-efficacy, patient trust in physicians, and participation in medical decision-making behaviors are included in the equation, there is statistical significance △*R^2^.* (△*R^2^* = 0.074 △*F* = 6.387, *P*<0.001; △*R^2^* = 0.096, △*F* = 92.790, *P* < 0.001; △*R ^2^*= 0.367, △*F* = 634.544, *P* < 0.001; △*R^2^* = 0.047, △*F* = 89.314, *P* < 0.001), **P*<0.05, ***P*<0.01, ****P* < 0.001; VIF_max_ = variance inflation factor maximum)

**Table S4.** *Examining the mediating effect with the Bootstrap method (standardized coefficients)*

| Paths | Effect type | S.E. | Effect sizes | Bias-corrected 95% CI | | | Percentile 95% CI | | | Supported hypothesis |
| --- | --- | --- | --- | --- | --- | --- | --- | --- | --- | --- |
|  |  |  |  | Lower | Upper | *p* | Lower | Upper | *p* |  |
| Trust in physicians  ® Satisfaction with medical services | Total effects | 0.033 | 0.771 | 0.710 | 0.822 | 0.001 | 0.712 | 0.824 | 0.001 | H_4a_ |
|  | Direct effects | 0.045 | 0.661 | 0.581 | 0.731 | 0.001 | 0.584 | 0.733 | 0.001 |  |
|  | Indirect effects | 0.020 | 0.110 | 0.078 | 0.146 | 0.001 | 0.077 | 0.144 | 0.001 |  |
| Self-efficacy  ® Satisfaction with medical services | Total effects | 0.018 | 0.055 | 0.030 | 0.091 | ＜0.001 | 0.027 | 0.087 | 0.001 | H_4b_ |
|  | Direct effects | / | / | / | / | / | / | / | / |  |
|  | Indirect effects | 0.018 | 0.055 | 0.030 | 0.091 | ＜0.001 | 0.027 | 0.087 | 0.001 |  |

*Note.* S.E. = Standard Error; 95% CI = 95% Confidence Interval
